# Novel Small Molecules with Anti-Inflammatory and Anti-Angiogenic Activity in a Mouse Model of Oxygen-Induced Retinopathy

**DOI:** 10.3390/cells13161371

**Published:** 2024-08-17

**Authors:** Adam S. Dayoub, Eesha Acharya, Adnan Dibas, Harlan P. Jones, Suchismita Acharya

**Affiliations:** 1 AyuVis Research Inc., Fort Worth, TX 76107, USA; adayoub@ayuvis.com (A.S.D.);; 2 The North Texas Eye Research Institute, University of North Texas Health Science Center, Fort Worth, TX 76107, USA

**Keywords:** TLR2, TLR4, retinopathy of prematurity, anti-inflammatory, anti-angiogenic, cytotoxic immune cells, prevention, treatment

## Abstract

Retinopathy of prematurity (ROP) has a dual-phase disease pathology; in phase 1, hyperoxia-induced vaso-obliteration occurs in the retinal vasculature due to increased oxidative stress (OS) and inflammation, followed by phase 2, where hypoxia increases the overproduction of growth factors, inducing retinal neovascularization. Toll-like receptor 2 and -4 (TLR2 and TLR4) overactivation, hyper-inflammation, macrophages, and neutrophil infiltration contribute to the developing ROP. AVR-121 and AVR-123 are novel classes of small-molecule dual inhibitors of TLR2/4 tested in a human leukemia monocytic cell line (THP-1) and cord-blood-derived mononuclear cells (CBMCs). Both compounds inhibited TLR2/4 signaling-related inflammatory cytokines in THP-1 cells and inhibited VEGF-induced neovascularization in human retinal endothelial cells (HRECs), which are hallmarks of ROP. In an oxygen-induced retinopathy (OIR) murine model, the intraperitoneal injection of AVR-123 in the hyperoxia phase (P7–P12) or a nanosuspension eyedrop of AVR-123 in the hypoxic phase (P12–P17) significantly reduced vaso-obliteration, angiogenesis, and inflammatory cytokine profiles while not inhibiting the necessary growth factor VEGF in the juvenile mouse eyes. The results are consistent with our hypothesis that targeting the dual TLR2/4 pathway will reduce inflammation, angiogenesis, and vaso-obliteration in vitro and in vivo and reduce cytotoxic immune cells. AVR-123 has the potential to be developed as a therapy for ROP.

## 1. Introduction

Retinopathy of prematurity (ROP) is a neurovascular disease propagated by abnormal retinal vascularization, primarily in preterm infants [1]. ROP affects 30–50% of preterm infants (<30 weeks gestational age, (GA) with a birth weight (BW) < 1500 g) [2]. ROP is one of the leading and preventable causes of childhood vision loss worldwide, affecting approximately 50,000 infants a year with irreversible vision impairment [3,4]. ROP has a profound financial impact on families, depending on the severity of the disease; current estimates per child are projected to range from USD 30,000 to 224,295 annually, including examination, treatment, and care [5]. ROP also leads to late sequelae in adolescence and adulthood by causing refractive changes, cataracts, glaucoma, tractional and exudative retinal detachment, and hemorrhage [6]. These late-stage conditions add to the overall cost of treating the disease and affect the entire life span of those born prematurely with ROP.

**Scheme 1:**
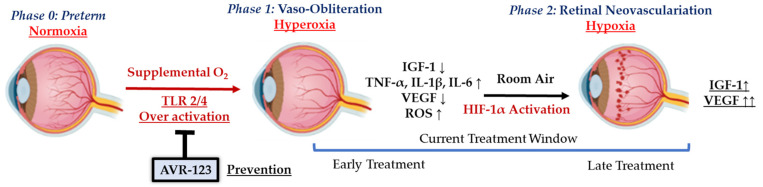
AVR-123 inhibits both TLR 2 and 4 stimulations, potentially preventing the development of ROP in premature eyes. TLR 2/4 is responsible for signaling the production of inflammatory cytokines such as TNF-α, IL-1β, IL-6 and iNOS. These cytokines produce Ph-1 effects in ROP (vaso-obliteration) and further drive HIF-1α, which increases VEGF and IGF-1, leading to Ph2 ROP. AVR-123 inhibits TLR 2/4 and controls the hyperactivation of the innate immune response, preventing vaso-obliteration and modulating downstream signaling to allow a normalized VEGF response, inhibiting IGF-1 and abnormal neovascularization.

ROP is a multifactorial disease with short gestational periods and can only be detected at 30–32 weeks of GA, at which point the disease has already progressed. Induced hyperoxia/hypoxia for preterm infants due to mechanical ventilation used for underdeveloped lungs is the leading cause of ROP development [7]. Human retinal vasculature development usually occurs between the 16th and the 40th week of gestation. Because of this defined timeline, preterm infants born <30 weeks GA have an incomplete peripheral avascular zone and incomplete retinal vasculature development [8]. ROP is the result of the disruption of retinal vasculature development in a biphasic modality: hyperoxic Phase-1 (Ph1) ROP, which presents retinal microvascular degradation or vaso-obliteration, and hypoxic Phase-2 (Ph2), which results in retinal ischemia triggering an overabundance of vascular endothelial growth factor (VEGF) and insulin growth factor-1 (IGF-1), leading to abnormal intravitreal neovasculature [9]. These phases are shown in Scheme 1.

The intravitreal injection of an anti-VEGF antibody is the only approved therapy for treating ROP and is a Ph2-only treatment after ROP diagnosis. However, anti-VEGF antibodies such as Avastin and Lucentis show moderate success, with high recurrence rates and late-stage reactivations that require surgical or laser interventions [10,11]. Additionally, the intravitreal route of dosing produces side effects such as eye-floaters, vision deficits, retinal detachment, blindness, and possibly death due to thromboembolic events caused by VEGF-induced increase in nitric oxide in the patient’s plasma [12,13,14]. In late-stage ROP, repetitive surgical treatment is needed once retinal detachment occurs [15,16,17]. Furthermore, ROP reactivation is prevalent after treatment with anti-VEGF drugs; therefore, meticulous arrangements for ongoing eye examinations after anti-VEGF therapy are critical in identifying and managing cicatricial ROP and possible retinal detachment with vision loss. Although VEGF and laser therapy for ROP treatment have succeeded in helping individuals, they each have unique risks and, most importantly, do not prevent or treat the condition directly; rather, they alleviate the symptoms.

A hyperoxia environment caused by a mechanical ventilator and an underdeveloped immune system in premature infants are major driving forces in the development of Phase 1 and Phase 2 ROP. Due to the imbalance in a neonate’s immune system, they are more susceptible to abnormal growth and pathological retinal angiogenesis than a term infant [18,19,20]. In the last ten years, multiple studies have identified neonatal inflammation as a critical modulator in the development and progression of ROP [21,22]. Dendritic cells (DCs) are reduced in neonatal peripheral blood, impairing T helper cells (Th1 and Th2) in combination with IL-10, leaving DC in a state of immaturity [23,24]. The cytokines interleukin-6 (IL-6), interleukin-1 beta (IL-1β), tumor necrosis factor-alpha (TNF-α), and angiogenic mediators like insulin growth factors are directly related to ROP. Neonate monocytes producing these cytokines have reduced capabilities regarding adhesion and infiltration into inflamed tissues, as in ROP [25]. TLR2 and TLR4 stimulation in neonate monocytes can facilitate a robust IL-6, TNF-α, and interleukin-10 (IL-10) response. However, unlike the adult counterpart, neonatal whole blood has a reduced response to stimulation by the TLR4 agonist LPS compared to stimulation by the TLR2 agonist peptidoglycan [26]. Neutrophils, which provide the first line of defense, are significantly impaired in neonates regarding their adhesion capabilities and impairment in making neutrophil extracellular traps (NETs) [27,28]. The neonatal immune imbalance suggests inhibiting the ROP-inducing cytokines IL-6, TNF-α, and IL-1β and immunomodulating neonatal immune cell types for an ROP-preventative treatment.

Systemic immune inflammation cascades, and the infiltration of activated macrophages in retinal blood circulation leads to the activation of TLR2 and TLR4 signaling in the ocular environment. The activation of TLR in resident macrophages/microglia, retinal pigment epithelial (RPE) cells, and endothelial cells perturbs the normal development of retinal vasculature and function in premature eyes, leading to ROP. We hypothesized that decreased inflammation leads to decreased cellular dysfunction and appropriate growth factor production, reducing vaso-obliteration and neovascularization. Altogether, the composite outcome would be a reduction in the degree of retinopathy in the eyes.

**Figure 1:**
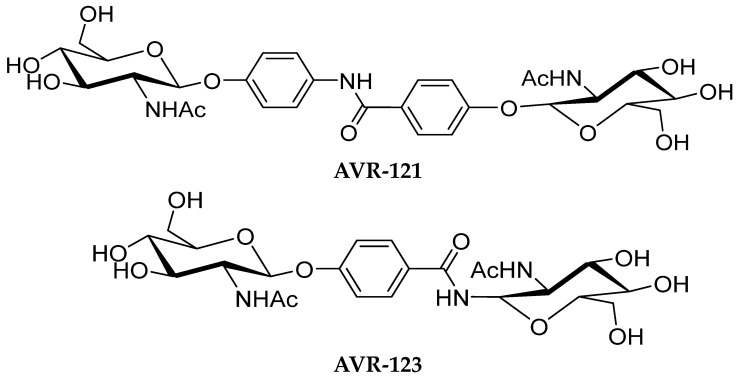
Chemical structures of AVR-121 and AVR-123.

We designed and synthesized two first-in-class novel compounds, AVR-121 and AVR-123. These synthetic chitin derivatives possess two molecules of N-acetyl glucosamine joined by a hydrophobic linker, as shown in Figure 1. The genesis of these analogs was based on modifying the chemical structure of chitohexaose, which was previously shown to be a potent TLR4 inhibitor with anti-inflammatory activities in a systemic endotoxemia mouse model [29]. The physicochemical properties and preliminary receptor-ligand docking study are shown in Supplemental Table S1 and Figure S1.

In the present study, we demonstrate the role of compounds AVR-121 and AVR-123 in modulating TLR2- and TLR4-mediated immune responses in human blood-derived mononuclear cells and the anti-angiogenic effect in retinal endothelial cells in vitro and in a murine model of OIR in vivo. We also developed a poly co-lactic glycolic acid (PLGA)-encapsulated nanosuspension formulation of AVR-123 with a sustained drug release profile. AVR-123 as a solution and a nanosuspension form were evaluated in the mouse OIR model by either systemic or eyedrop dosing, respectively, and retinal vaso-obliteration and angiogenesis were assessed. The changes in cytokines and growth factors were evaluated in the mouse retina, and the immune cell changes were determined in the mouse spleen to understand the immunomodulatory activity of AVR-123.

## 2. Materials and Methods

### 2.1. Chemicals, Reagents, Media, and ELISA Kits

The synthesis and structural characterization of compounds AVR-121 and AVR-123 and the PLGA-encapsulated AVR-123 were conducted in the laboratory of AyuVis Research Inc. (Fort Worth, TX, USA), following the in-house procedures [30]. Endotoxin-free phosphate-buffered saline (PBS) was purchased from Sigma-Aldrich Inc., St. Louis, MO, USA. RPMI-1640 medium (Cat# 11875093) was purchased from GIBCO, Waltham, MA, USA, and fetal bovine serum (FBS, Cat# S12340H) was purchased from BioTechne, Minneapolis, MN, USA. Endothelial cell growth supplement medium (Cat #39-059-9) was purchased from R&D Systems, Minneapolis, MN, USA. 3-(4,5-dimethylthiazol-2-yl)-2,5-diphenyl-2H-tetrazolium bromide (MTT) proliferation assay reagent (Cat # G3580) was purchased from Promega, Madison, WI, USA. Phorbol myristyl acetate (PMA) (Cat# P1585, Sigma, St. Louis, MO, USA), LPS (Cat # tlrl-smlps, InvivoGen, San Diego, CA, USA), PAM_3_CSK_4_ (Cat# 4633, Tocris, Bristol, UK), TAK-242 (Cat# A3850-10, APExBIO, Houston, TX, USA), Alexa Fluor 594-conjugated isolectin GS-IB4 (Cat # I21413 Invitrogen, Waltham, MA, USA), and VEGF (Cat # 100-20-10UG, ThermoFischer, Waltham, MA, USA) were purchased. The following ELISA kits were purchased from Raybiotech, Peach Corners, GA, USA: Cat # ELH-TNF-a, tumor necrosis factor-alpha (TNF-α); Cat # ELH-IL1b, interleukin-1 beta (IL-1β); Cat # ELH-IL6, interleukin-6 (IL-6); Cat # ELH-TLR2, TLR2; and Cat # ELH-TLR4, TLR4. For in vitro angiogenesis assay, the Tube Formation kit (Cat#3470-096-K) was purchased from BioTechne, Minneapolis, MN, USA.

### 2.2. Formulations of AVR-121 and AVR-123

AVR-123 was reconstituted in 0.9% sterile normal saline for cell assay and mouse efficacy studies to provide a final dose concentration. For cell studies, the doses were 1, 10, and 100 µM, prepared freshly. For animal studies, 10 mg/kg as a colorless solution was injected with IP (30 µL) daily from P7 to P12. The PLGA nanoparticles encapsulated with AVR-123 were resuspended in deionized sterile water to create a nanosuspension with a final dose concentration of 0.11 mg/kg/eyedrop (5 µL).

### 2.3. Animals

Female pregnant C57BL/6 J mice were purchased from The Jackson Laboratory (Bar Harbor, ME, USA) and were maintained in a breeding colony at the University of North Texas Health Science Center (Fort Worth, TX, USA) Animal Care Facilities with Animal Welfare Assurance No. D16-00417 (A3711-01). Animal procedures were performed according to the NIH Guidelines for the Care and Use of Laboratory Animals. They were approved by the Institutional Animal Care and Use Committee (IACUC) of UNTHSC, TX, USA (Protocol no. 2020-0002). Neonatal mouse pups (both males and females) born from female pregnant mice were used for the OIR study.

### 2.4. Cell Proliferation Assay

Human peripheral blood mononuclear cells (hPBMCs) (Cat # 70025.3, STEMCELL Technologies, Cambridge, MA, USA) were cultured at cell density (1 × 10^5^) in RPMI-1640 medium and FBS in 5% CO_2_ at 37 °C. Cells were dosed with either AVR-121 or AVR-123 in ascending concentrations (0.1 µM, 1.0 µM, 10 µM, and 100 µM) and incubated for 24 h. Human retinal microvascular endothelial cells (HRECs) (Cat# ACBRI 181, Cell Systems, Kirkland, WA, USA) were cultured and treated with 100 mM of either AVR-121 or AVR-123 and incubated for 24 h. An MTT cell viability/proliferation assay was performed, and absorbance was read at 490 nm on a Cytation5 (Gen5 3.12, BioTek Instruments, Winooski, VT, USA). The percentage of viable cells was calculated from the change in absorbance in the AVR-compound-treated cells compared to untreated control cells. N = 4, * *p* < 0.05, ** *p* < 0.001. One-way ANOVA. GraphPad Prism 10.0.

### 2.5. Cytokines, TLR2 and TLR4 Protein Quantification Using ELISA

THP-1 leukemia cells (Cat# TIB-202, ATCC, Manassas, VA, USA) were cultured in 24-well plates at the cell density 1 × 10^5^ in RPMI-1640 medium and FBS and stimulated with PMA (200 ng/mL) for 24 h in 5% CO_2_ at 37 °C. Cells were treated with PBS and different drug concentrations, as follows: AVR-121 (10 µM and 100 µM), AVR-123 (10 µM and 100 µM), LPS (25 ng/mL), and PAM_3_CSK_4_ (50 ng/mL). Supernatants were used for assessing the concentrations of TNF-α and IL-1β, and cell lysates were used to quantify TLR2 and TLR4 protein concentrations via ELISA assay. The plates were read using a BioTek Synergy plate reader. N = 4, * *p* < 0.05, ** *p* < 0.001. We used one-way ANOVA, with GraphPad Prism 10.0.

Human cord blood mononuclear cells (CBMCs) were purchased from Cat# CBMNC015C, CGT Global, Folsom, CA, USA. They were cultured at the cell density 3 × 10^6^ in 6-well plates in RPMI-1640 medium and FBS for 24 h in 5% CO_2_ at 37 °C. Cells were treated with either AVR-123 (100 µM), LPS (50 ng/mL), or TAK-242 (1 µM). Cytokine analysis was performed for TNF-α, IL-1β, and IL-6 via ELISA assay and read on a BioTek Synergy plate reader. N = 3, * *p* < 0.05, ** *p* < 0.001. We used one-way ANOVA, with GraphPad Prism 10.0.

### 2.6. In Vitro Tube Formation Assay

An in vitro angiogenesis assay tube formation (Cultrex) kit was utilized per manufacturer recommendations. Briefly, 60,000 human retinal microvascular endothelial cells (HRECs) (Cat# ACBRI 181, Cell Systems, Kirkland, WA, USA) were seeded in fresh endothelial cell growth medium supplemented with endothelial cell growth supplement (ECGM) (R&D Systems #39-059-9) overnight. BME solution (provided in the kit) without phenol red was prepared on ice and incubated at 37 °C for 1 h to gel. HRECs were incubated with Calcein AM solution for 30 min before harvesting. Cells were removed with Accutase, washed with PBS, and seeded in aliquots for dosing on BME gel. Cell suspension aliquots (~60,000 cells) were placed in ECGM containing the following: no drug + ECGM, VEGF (100 ng/mL) + ECGM as control, VEGF + AVR-121 (100 µM) + ECGM, and VEGF + AVR-123 (100 µM) + ECGM. Cells were incubated in drug solutions for between 4 and 8 h in 5% CO_2_ at 37 °C and visualized via epi-fluorescence microscopy. N = 4.

### 2.7. In Vitro Cell Migration Assay

In total, 30,000 HRECs/well were seeded into 12-well tissue culture plates and were growth-arrested by incubating in a serum-free medium overnight for 24 h. Using a 200 µL pipette tip, a 0.5 mm gap was made in each well, and images were captured using a phase contrast microscope. Cells were then co-treated with VEGF (100 ng/mL), and the test compounds AVR-121 or AVR-123 were combined. All groups were refreshed with treatment reagents every 12 h. The wounds were visualized directly through a light microscope at 0 h and 24 h, and photomicrographs were acquired. A representative photomicrograph is shown at the magnification of 4×.

### 2.8. Murine Oxygen-Induced Retinopathy Study

C57BL6/J mouse pups (males and females) were subjected to hyperoxia (75%) in a hyperoxia chamber (BioSpherix Pro-Ox Model 110, Parish, NY, USA) at P7–P12 and then transferred back to a normal oxygen atmosphere. In experiment number one, P7–P12 mice (*n* = 3) were injected via intraperitoneal injection (IP) with AVR-123 (10 mg/kg) once daily for 5 days. In experiment number two, P12–P17 mice (*n* = 5) were treated with AVR-123 nanosuspension eyedrops (1%) and 10 mg/kg IPs once daily for 5 days. Blank nanoparticles were instilled as nanosuspension eyedrops in control mouse eyes subjected to hyperoxia. Mice were sacrificed on P18 in both experiments. For the qPCR experiment to assess the mRNA changes from the retina, *n* = 3 mouse pups were used.

### 2.9. Lectin Staining and Flat Mount in Murine Retinas

C57BL6/J mouse eyeballs were fixed in 4% paraformaldehyde (Electron Microscopy Sciences, Hartfield, PA, USA) for 15 min. Anterior segments were removed, and eyecups were post-fixed with 4% paraformaldehyde for 24 h. Retinas were dissected and permeabilized overnight with 0.3% Triton X-100 (Sigma) in PBS with overnight staining with Alexa Fluor 594-conjugated isolectin GS-IB4 (5 µg/mL). Retinas were flattened on microscope slides with four incisions from the ora serrata to the equator. The flat mounts were observed by epi-fluorescent microscopy (Olympus Life Sciences, Center Valley, PA, USA). Retinal neovascularization was measured in Adobe Photoshop, 7.0 and the degree of vaso-obliteration was quantified using Image J 1.54f software (NIH). *n* = 3–5 retina.

### 2.10. qPCR Analysis of Mouse Retinas

Mouse retinas from the control and treated groups were collected on day 18 from experiment 2 (eyedrop dosing). The mRNA levels for IL-1β, IL-6, IL-10, TNF-α, iNOS, VEGF, TGFβ2, and IGF-1 were measured by qPCR, and data were normalized to the β-actin control. *n* = 3. The primers used are listed in Table 1 below.

### 2.11. Flow Analysis

Mouse spleen cells collected on day 18 were pre-incubated with FcBlock 1 µg/10^6^ cells before staining with the following fluorescent conjugate cell antibodies at specified concentrations in the dark at room temperature, as shown in Table 2. Two-color parameter flow cell staining was performed on all samples. Single-cell staining was performed to set the compensation compared to no-stain cells. Data were acquired using the Cytek Aurora 4-laser flow cytometer (Cytek, Fremont, CA, USA) and analyzed using FlowJo V 10.8.1

### 2.12. Statistical Analysis

Statistical analysis was performed using GraphPad Prism version 10.2.1; Tukey’s (two-way ANOVA) and Dunn’s multiple comparison test (one-way ANOVA) or the Mann–Whitney test (*t*-test) was used. All data presented are the mean ± standard error of the mean (SEM) from at least three biological replicates and two technical replicates per treatment group. Values of *p* < 0.05 were considered statistically significant. * *p* < 0.05, ** *p* < 0.01, *** *p* < 0.001, **** *p* < 0.0001.

## 3. Results

### 3.1. AVR-121 and AVR-123 Are Not Cytotoxic to Human Peripheral Blood Mononuclear Cells and Human Retinal Endothelial Cells (HRECs)

**Figure 2:**
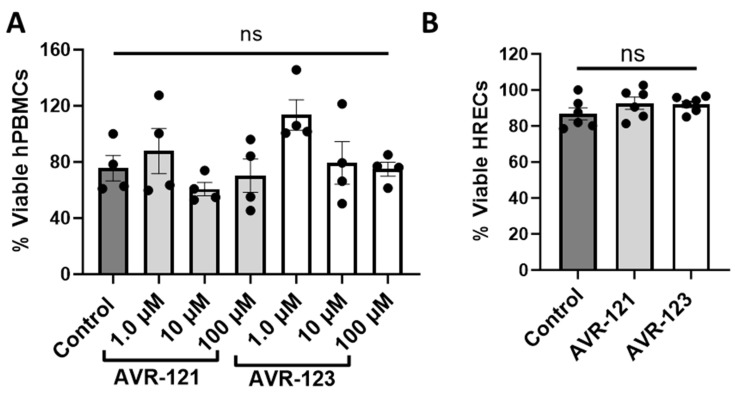
Cell viability assay. (**A**) In hPBMCs after 24 h of AVR-121 and AVR-123 treatment (Promega), both compounds showed no cytotoxicity up to 100 µM concentration, and the percentage of viable cells was comparable to control cells. At 1.0 µM concentration, increased cell proliferation or metabolic activity was observed, but this was not statistically significant (ns). (**B**) No cytotoxicity was observed in HRECs, N = 3–4.

Our first aim was to establish that AVR-121 and AVR-123 are not cytotoxic to human peripheral blood mononuclear cells (hPBMCs), as these cell types are a primary responder to innate immune insults triggered by microbial or hyperoxic oxidative stress (OS) insults. As shown in Figure 2A,B, we performed an MTT cell viability assay with a range of concentrations (1 µM, 10 µM, and 100 µM) for both AVR-121 and AVR-123 treated for 24 h. Next, we tested 100 µM of either AVR-121 or AVR-123 on HRECs.

After 24 h, we observed no significant change in the percentage of cell viability after treatment with either AVR-121 or AVR-123 compared to the control group in both hPBMCs and HRECs at all treated doses.

### 3.2. Treatment of AVR-121 and AVR-123 Inhibited Inflammatory Cytokines Via Toll-Like Receptor-2 and -4 Inhibition In Vitro

**Figure 3:**
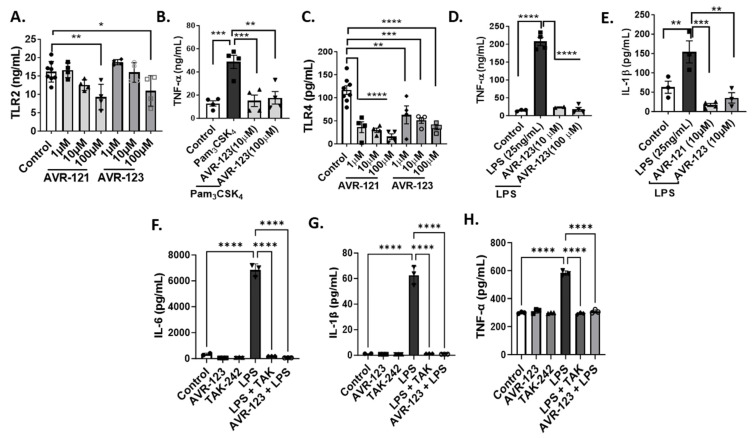
Receptor inhibition and cytokine assays. Briefly, compounds AVR-121 and AVR-123 (1, 10, and 100 µM) were dosed with PMA (200 ng/mL)-activated THP-1 cells and were incubated for 48 h. (**A**,**C**) TLR2 and TLR4 protein levels in cell lysate were probed via ELISA. (**B**,**D**,**E**) Activated THP-1 cells were treated with either LPS or PAM_3_CSK_4_ or in combination with AVR-121 and AVR-123 for 48 h, and cytokines TNF-α and IL-1β were assessed in the supernatant via ELISA. (**F**–**H**) CBMCs were treated with AVR-123 (100 µM), LPS, and TAK-242 (TAK) for 24 h and assessed for cytokines IL-6, IL-1β, and TNF-α in the cell supernatant via ELISA. N = 3, data are presented as mean ± SEM, * *p* < 0.05, ** *p* < 0.01, *** *p* < 0.001, **** *p* < 0.0001. One-way ANOVA, Graph Pad Prism.

Next, we performed in vitro studies using THP-1 human monocytic cells to determine the TLR2 and TLR4 inhibitory activities of the compounds AVR-121 and AVR-123 and their regulation of inflammatory cytokine production. Using a known TLR2 agonist, PAM_3_CSK_4_ (Figure 3A,B), and TLR4 agonist, LPS (Figure 3C–E), we demonstrated that both AVR-121 and AVR-123 at 10 and 100 µM doses significantly reduced TLR2 protein in activated THP-1 cells in a dose-dependent manner compared to the control and reduced the Pam_3_CSK_4_-induced TNF-α concentration. Similarly, both compounds dose-dependently reduced the TLR4 protein concentration beginning at 1 µM concentration (Figure 3C) and significantly reduced LPS-induced IL-1β and TNF-α, as shown in Figure 3D,E. In addition, we used human cord blood mononuclear cells (CBMCs), as shown in Figure 3F–H, to demonstrate that treatment with 100 µM of AVR-123 significantly reduces the LPS-induced inflammatory cytokines TNF-α, IL-1β, and IL-6, and the anti-inflammatory activity is comparable to a commercial TLR4 inhibitor, TAK-242.

### 3.3. AVR-123 Inhibits VEGF-Induced Vascular Tube and Microcapillary Formation

**Figure 4:**
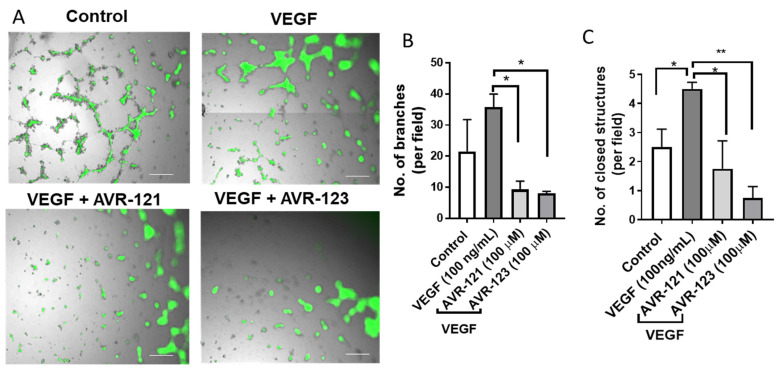
Evaluation of anti-angiogenic activity in human retinal endothelial cells (HRECs) following Matrigel tube formation assay. (**A**) Micrograph images showing new tube formation after Calcein-AM labeling of the cells 6 h post-treatment. Scale bar = 1000 µm (**B**) The number of closed structures or new tubes increased in VEGF-treated cells, and VEGF+AVR-121 or AVR-123 treatment significantly reduced them. (**C**) Branched microcapillaries increased after VEGF treatment and decreased after AVR-121 and AVR-123 treatment. N = 4–6, * *p* < 0.5, ** *p* < 0.05, one-way ANOVA.

Human retinal endothelial cells (HRECs) were used to demonstrate the effect of AVR-123 on neovascularization in an in situ model (Figure 4). TLR2 signaling in the retina is associated with neovascularization in mice, as reported using a TLR2 knock-out model [31]. Inflammation contributes to the activation of angiogenesis and is partially mediated through the TLR2/VEGF retinal signaling pathway. As shown in Figure 4A, we anticipated that inhibiting TLR2 signaling would reduce angiogenesis and new tube formation. VEGF treatment on HRECs showed an almost 4-fold increase in branches and closed vascular structures, indicating increased vasculogenesis and angiogenesis. However, after treatment with either the compound AVR-121 or the compound AVR-123 (100 µM) to the VEGF-treated cells, the number of branches and closed structures was significantly reduced. AVR-123 was found to be more potent (~2-fold) than AVR-121 in this assay and, therefore, was selected to be tested further in the in vivo model.

### 3.4. AVR-123 Inhibits VEGF-Induced Endothelial Cell Migration

**Figure 5:**
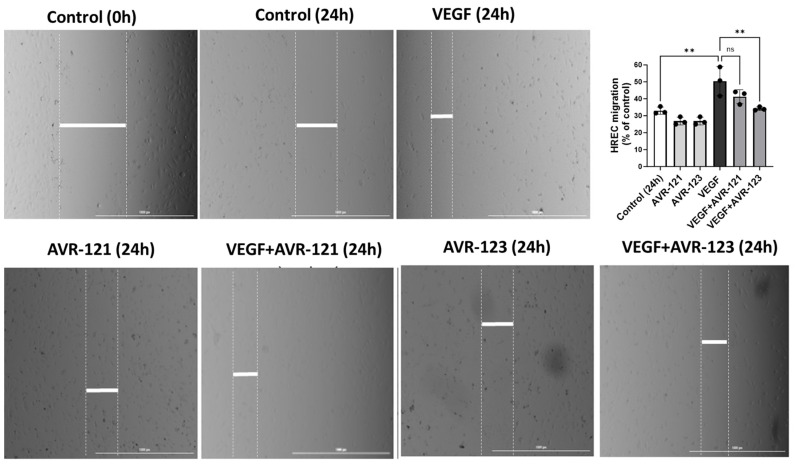
Evaluation of cell migration and wound closure activity in human retinal endothelial cells (HRECs) following scratch formation assay. Cells were seeded and allowed to attach to tissue culture plates. For the cell migration study, micropipette tips were used to make scratch lines on wells, and images were taken to measure the distances of gaps. Cells were treated with either VEGF (100 ng/mL) or 100 µM of AVR-121, AVR-123, or a combination of both and incubated for 24 h. Cell migration was imaged and analyzed to assess the final distances of gaps via ImageJ, and EC migration was calculated as % of control. N = 4, ** *p* < 0.01. One-way ANOVA. Scale bar = 1000 µm. ns: not significant.

During pathogenic angiogenesis, as in the ROP, the neovascular growth leads the microcapillaries to migrate, breach the RPE tight junction, and travel to the retina and vitreous. Using the micropipette-induced in vitro scratch assay in HRECs to determine the % for cell migration and wound closure, we demonstrated that, while VEGF (100 ng/mL) induced significant cell migration and scratch gap (wound) closure after 24 h, as shown in the bar graph (Figure 5), VEGF+AVR-123 (100 µM) reduced the cell migration. AVR-121 or AVR-123 alone had no significant effect on cell migration or wound closure.

### 3.5. Formulation Development for AVR-123

**Figure 6:**
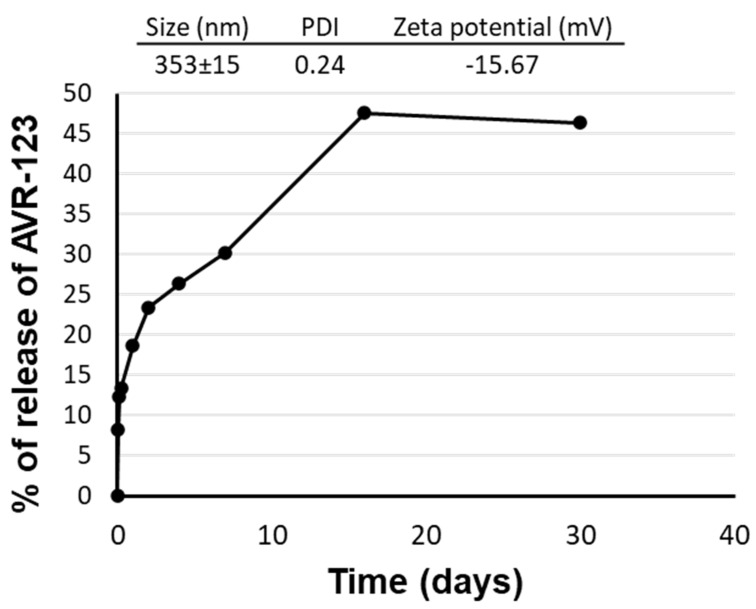
AVR-123 PLGA nanosuspension characterization and drug release profile.

To avoid invasive intravitreal injection and facilitate the passive diffusion of the drug through the cornea to the scleral route to reach the posterior segment of the eye, we developed a nanosuspension formulation for topical ocular eyedrop dosing. Following our previously published protocol, [32,33,34]. AVR-123 was encapsulated in an FDA-approved biodegradable polymer poly-(lactic-co-glycolic) acid (PLGA) and polyvinyl alcohol (PVA). The size of the nanoparticle was found to be <400 nm, with an encapsulation efficacy of 57%. The compound AVR-123 slowly releases in PBS7.4 from the nanoparticle with a burst release of 20–25% over 12–24 h and a cumulative release of ~50% of AVR-123 at 37 °C at 30 days, as shown in Figure 6. Our previous publications demonstrated that small molecules with similar physicochemical properties to AVR-123, when encapsulated in this PLGA vehicle, were bioavailable to the vitreous humor (VH), retina, choroid, and optic nerve after single-eyedrop dosing in mouse and rat eyes [32].

### 3.6. AVR-123 Reduced Vaso-Obliteration and Angiogenesis in a Murine Oxygen-Induced Retinopathy (OIR) Model

**Figure 7:**
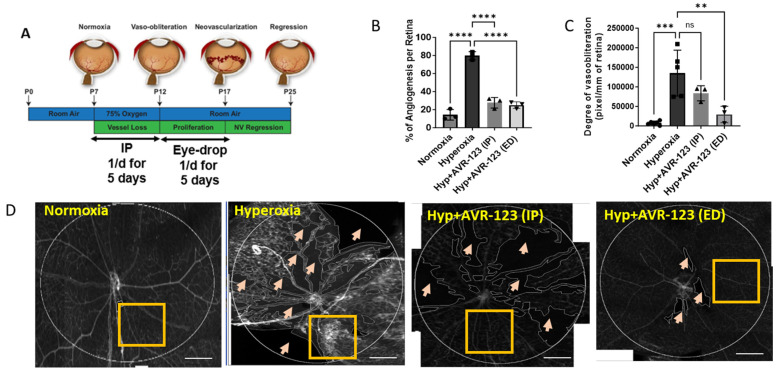
AVR-123 reduces vaso-obliteration and angiogenesis in OIR murine retinas. (**A**) AVR-123 was dosed once a day via IP (10 mg/kg) from P7–P12 and with AVR-123 (ED) (1% nanosuspension) for five days from P12–P17. At P18, both groups of mouse pups were sacrificed, and retina flat mounts were prepared and stained with isolectin. (**B**) AVR-123 (IP) and AVR-123 (ED) significantly reduced % of angiogenesis and neovascular tufts. (**C**) AVR-123 (IP) and AVR-123 (ED) significantly reduced the degree of vaso-obliteration quantified using Image J software. (**D**) The retina from both experiments was stained with isolectin to visualize the blood vessels, neovascular tufts (yellow squares), and obliterated vasculatures in the retina (arrows). Scale bar = 1000 µm**,** *n* = 3–5/group. ** *p* < 0.01, *** *p* < 0.001, **** *p* < 0.0001, one-way ANOVA. ns: not significant.

Mouse pups on day P7 were introduced to a high-oxygen atmosphere of 75% O_2_, as shown in Figure 7A. AVR-123 was formulated into a 1% PLGA-loaded nanosuspension eyedrop [AVR-123(ED)], as well as a 10 mg/kg solution in PBS [AVR-123(IP)]. P7 mice were given intraperitoneal injections (IPs) once a day for five days until P12, and, for Expt-2, AVR-123 nanosuspension eyedrops were administered once a day from P12–P17 (hypoxic phase). The rationale behind administering the drug via IP was twofold: firstly, it was experimentally difficult to deliver eyedrops in P7 mouse pup eyes, and secondly, we hypothesized that AVR-123 could inhibit retinal inflammation by reducing systemic inflammation and may be bioavailable to the retina through the central retinal artery. As shown here, in retinal flat mounts stained with isolectin to visualize blood vessel structures (Figure 7B–D), extensive vaso-obliteration of the retinal vasculature (arrows) was observed in hyperoxic retinas compared to the normoxic mouse retinas. Our data demonstrate that when mouse pups’ eyes are treated with AVR-123 either systemically or topically in both phases, the degree of angiogenesis decreases by >50% compared to the hyperoxic control retina (Figure 7B). However, the degree of vaso-obliteration was significantly reduced in the eyedrop-treated cohort (Figure 7C). The limitation of our study is that we have not assessed these changes at P12 after IP dosing.

### 3.7. AVR-123 Treatment Reduced Pro-Inflammatory Cytokines While Not Negatively Affecting the Essential Growth Factors for Retinal Vascular Development

**Figure 8:**
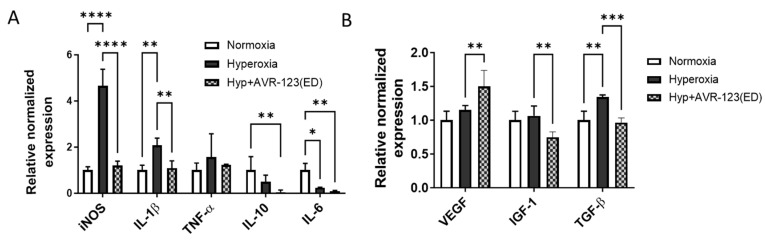
qPCR data from mouse retinas. Mouse retinas treated with AVR-123 (ED) for 5 days (P12–P17) were isolated at P18, and RNA was extracted, followed by cDNA synthesis. qPCR was performed to assess changes in the following genes: iNOS, TNFα, IL-1β, IL-6, and IL-10 (**A**), and VEGF, TGFβ2, and IGF-1 (**B**); β-actin was used for the housekeeping gene. *n* = 3. * *p* < 0.05, ** *p* < 0.01, *** *p* < 0.001, **** *p* < 0.0001, two-way ANOVA.

Here, in Figure 8, we demonstrated that AVR-123 significantly reduced inflammatory innate immune signaling molecules in the mice treated with AVR-123 NP eyedrops. Mouse pup retinas from the OIR models treated with 1% AVR-123NP eyedrops were used for RNA extraction and (real-time) qPCR to determine the mRNA levels of cytokines and growth factors. We probed for several pro- and anti-inflammatory molecules, including IL-6, IL-1β, iNOS, TNF-α, and IL-10, along with growth factors VEGF, IGF-1, and TGF-β2. There was a significant increase in iNOS, IL-1β, and TNF-α in the hyperoxic retinas, and, after treatment with AVR-123NP (ED), these genes were significantly downregulated (Figure 8A). IL-10 and IL-6 mRNA were significantly reduced compared to the normoxic retina. There was a significant increase in insulin growth factor-1 (IGF-1) and transforming growth factor beta-2 (TGFβ2) in the hyperoxic retinas, and after treatment with AVR-123 (ED), these genes were significantly downregulated (Figure 8B). Interestingly, VEGF mRNA was found to be considerably higher in AVR-123 (ED)-treated retinas, which is an essential growth factor for the development of the retinal vasculature, indicating that the mechanism of anti-angiogenic activity in AVR-123 is partially inhibiting the excess production of IGF-1 and TGFβ instead of VEGF. To confirm this, further experimentation is required to verify the quantity of the proteins using Western blotting.

### 3.8. Treatment with AVR-123 Reduces Populations of Macrophages, CD8^+^ T Cells, and Neutrophils in Mouse Pup Spleens from the OIR Model

**Figure 9:**
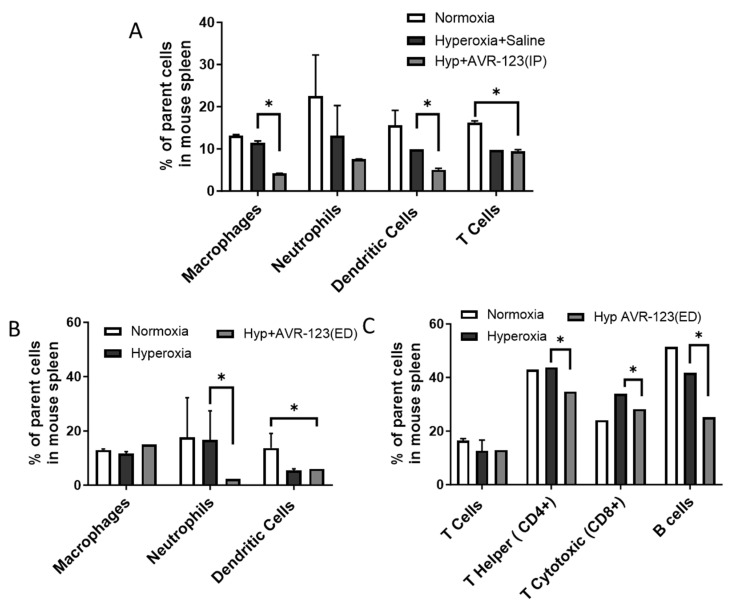
Effect of AVR-123 treatment on mouse splenic immune cells from the OIR mice. (**A**) The splenic immune cell populations from OIR mice injected IP with AVR-123 (P7–P12) were collected at P18, and the cells (*n* = 3 mouse spleens) were stained with immune cell-specific antibodies and analyzed via flow cytometry. (**B**,**C**) The splenic immune cell populations from OIR mice treated with AVR-123 (ED) during P12–P17. Treatment with AVR-123 reduced populations of macrophages (F4/80+CF11b+), CD8^+^ T cells (CD3+CD8+), neutrophils (Ly6G+CD11b+), and dendritic cells (CD11c+MHCII+) in mouse pup spleens compared to the hyperoxia mouse group. *n* = 3. * *p* < 0.05, *t*-test.

The results in Figure 9A show that AVR-123 (IP) treatment to mice during hyperoxia (P7–P12) reduced macrophages, neutrophils, dendritic cells, and T cells but did not fully ablate them, allowing immune cell communication to occur and proceed with retinal development via immunomodulation. The reduction in macrophage populations compared to hyperoxia mouse spleens demonstrated the capability of AVR-123 to reduce systemic macrophages. As shown in Figure 9B, we observed that AVR-123 NP eyedrops during the hypoxic phase (P12–P17) did not affect the % of macrophages in the spleen, possibly due to low systemic bioavailability. Cytotoxic CD8^+^ T cells are responsible for inflammatory signaling and migration to the retina and vasculopathy in oxygen-induced retinopathy [35]. CD8^+^ T cells have also been implicated as a developmental factor in ROP progression [36]. Here, we observed that AVR-123 NP eyedrops reduced the cytotoxic CD8^+^ T cell populations (Figure 9C). Lastly, the lymphocyte-to-monocyte ratio is directly associated with ROP development [37], and here, we showed that, by both the IP and ED routes of dosing during the hyperoxic and hypoxic phases, respectively, AVR-123 reduced lymphocytes such as dendritic cells, neutrophils, and B cells, compared to the hyperoxia groups. Neutrophils were significantly lowered in both experiments, as shown in Figure 9A,B, in contrast to the hyperoxia groups, which is salient, as a neutrophil-to-lymphocyte ratio is a risk factor for ROP treatment, as described in a 220-infant cohort clinical study [38]. We believe that the observed change in splenic immune cell populations after eyedrop dosing is possibly due to the systemic leakage of some of the drugs via the iridocorneal and lacrimal gland drainages that generally occur with eyedrop dosing. A pharmacokinetic study in plasma collected after ED dosing could confirm this observation.

## 4. Discussion

The present study suggests a small immunomodulatory molecule, AVR-123, as a novel preventative treatment for ROP that not only has the potential to reintroduce balance in homeostatic retina development in preterm infants but also provides an alternate strategy to invasive intravitreal injection and laser surgery by using an eyedrop delivery system. The main objective of this study was to assess the immunomodulatory and healing efficacy of AVR-123 in a cell milieu (in vitro) and an OIR mouse model. The OIR study that we reported here demonstrated that a PLGA-encapsulated nanosuspension of AVR-123 delivered as an eyedrop is appreciably effective in preventing Ph1 vaso-obliteration, as well as ameliorating Ph2 neovascularization in a murine retina, a hallmark of ROP.

TLRs, in particular, facilitate pattern recognition, initiate various immune cells’ activation in neonates, and are highly polymorphic and unique to preterm birth [39]. In the human eye, TLR2 recognizes peptidoglycan found in Gram-positive bacteria, leading to the hyperexpression of pro-inflammatory cytokines and growth factors such as IL-1, IL-6, IL-8, b-FGF, TGF-β1, and VEGF, as critical mediators of pathological angiogenesis [31,40]. In particular, TLR2 signaling in the retina leads to neovascularization, and inflammation-activated angiogenesis is partially mediated via TLR2/VEGF signaling [31]. Also, in the human eye, TLR4 is highly expressed in retinal tissues and microglial cells, suggesting its significant role in immune responses and modulation [41]. TLR4 activation is an essential contributor in neonatal diseases, including ROP, and retinal ischemia [42]. TLR4 is expressed on retinal pigment epithelial (RPE) cells and endothelial cells. It is a primary component of retinal microglial activation (resident macrophages in the retina) and promotes retinal inflammatory diseases [43]. Low TLR4 levels in retinal endothelial cells have demonstrated protection against retinopathies [44]. TLRs also participate in cytokine production from free radicals and reactive oxygen species (ROS) resulting from excessive oxygen in the bloodstream during the mechanical-ventilation-induced hyperoxia period in premature babies and play a key role in the development of ROP [45,46,47]. Heme-oxygenase-1 (HO-1) is an ally, as its activity rapidly rises due to free radicals [48]. In the hyperoxic (Ph1) stage of ROP, retinal mitochondria overproduce free oxygen radicals, which progress ROP [49]. Lastly, pathological neovascularization (Ph2) ROP can possibly be reduced by HO-1, contributing to hypoxia-inducible factor-1 alpha (HIF-1α) stabilization [50]. Because ROP involves altered endothelial cells, abnormal retinal vascularization, and the dysregulation of VEGF expression coupled with ROS-promoting inflammatory cytokines buildup via the activation of TLR2 and 4, targeting TLRs for VEGF realignment in ROP yields an additional favorable target in the prevention and treatment of the pathology [51].

Our primary in vitro study confirmed that AVR-123 inhibits the TLR2 agonist PAM_3_CSK_4_ and TLR4 agonist LPS-induced inflammatory cytokines TNF-α and IL-1β, as well as reducing TLR2/4 protein abundance. Additionally, we demonstrated this anti-inflammatory effect in both human monocytic THP-1 cells and human CBMCs, to increase the clinical translatability of these primary findings.

Wang et al. and Chen et al. have reported that tube formation assays are an excellent prognosticator of a drug’s effect on angiogenesis and repair [52,53]. Using HRECs in in situ models, AVR-121 and AVR-123 inhibited VEGF-induced tube formation, angiogenesis, and cell migration. TLR2 activation in the retina of WT mice has been reported to increase neovascularization compared to a TLR2 knock-out model. Inflammatory molecules, including TGF-β and IL-6, contribute to the increased neovascularization and are partially mediated through the TLR2/VEGF retinal signaling pathway [31]. Our data suggest that, by inhibiting TLR2 signaling, AVR-123 reduces IL-6 and TGFβ but does not act as a direct VEGF receptor antagonist, unlike reported small-molecule receptor tyrosine kinase (RTK) inhibitors used for anticancer therapy [54,55]. Moreover, the complete inhibition of VEGF in a developing eye is deleterious, and our mouse retina qPCR data show that AVR-123 moderately increases VEGF and allows physiological vascular growth in the mouse eye. At the same time, it significantly inhibits other undesired growth factors, IGF-1 and TGF-β2, which are upregulated in hyperoxic mouse retinas. This is a unique and selective approach offered by AVR-123, which needs further evaluation.

It should be noted that the currently used FDA-approved therapy does not mechanistically prevent ROP pathology; it only targets the pathways after ROP occurs by blocking a specific cascade protein marker. Eyedrop formulations using Rho-kinase inhibitors, including Fasudil 0.5% eyedrops, ripasudil 0.4% eyedrops, and β-blocker Propranolol 0.2% eyedrops, are being studied in clinical trials for ROP. Potential pitfalls with these eyedrops include optimal administration timing that may exacerbate the condition if given too early. Mechanistically, they are not beneficial in preventing vaso-obliteration (Ph1) and are only helpful during the proliferative phase (Ph2) [56,57]. Compound AVR-123 is encapsulated in PLGA nanoparticles (NPs) to release the drug slowly and for longer, to avoid more than one dose per day. AVR-123 NP (ED) significantly decreased vaso-obliteration and neovascularization after eyedrop administration.

The use of AVR-123 NP eyedrops with no observed side effects in our mouse OIR model may also augment the standard of care intravitreal injection of anti-VEGF antibody therapy or laser surgery. Most importantly, the cost for families and the potential damages to neonate’s eyes could significantly drop, which remains to be demonstrated. In addition to AVR-123’s anti-angiogenic effect, we demonstrated that AVR-123 reduced inflammatory and ROP-specific cytokines in the OIR mouse retina, such as iNOS and IL-1β, compared to hyperoxic retinas. The inhibition of IL-6 and IL-1β is essential, as they are predominantly responsible for innate immune inflammation and lead to a worse ROP prognosis [58,59]. Also, the inhibition of excess iNOS expressed in the avascular retina reduced retinal neovascularization [60,61,62] and retinal neuronal cell death [63,64]. Altogether, the composite outcome is a decrease in the degree of retinopathy in neonatal eyes.

This study also reported that AVR-123 is not simply an inhibitor of TLR2 and TLR4 but demonstrates immunomodulation that segues into a complete immune response across innate to adaptive immunity. Our finding demonstrated AVR-123’s efficacy in reducing multiple splenic immune cell types, such as macrophages, neutrophils, dendritic cells, and cytotoxic CD8^+^ T cells, implicated in ROP pathology. Devy et al., for example, reported that a reduction in CD8^+^ T cells was needed for significant treatment in ROP [35]. Also, Qiu et al. reported a dendritic cells increase in OIR mouse models of ROP, and a reduction in these cells could be beneficial in treatment [65]. Systemic macrophages are the immune cells responsible for inflammatory cytokine production, such as IL-6, TNF-α, and IL-1β, which can activate the retinal endothelium cells, activating microglia/macrophages [66], and the inhibition of systemic macrophages can reduce ROP pathology [67]. AVR-123, delivered systemically via IP, appreciably reduced macrophage populations in the mouse pup spleen. This is salient to ROP treatment, as systemic macrophage populations have been shown to cross-talk with retinal microglia cells and can directly influence microglia M1 states in the central nervous system and, by definition, retinas [68].

Additionally, we report that AVR-123 (ED) reduces cytotoxic CD8^+^ T cell populations, which is a salient finding, as CD8^+^ T cells are responsible for inflammatory signaling to the retina, vasculopathy in OIR, and potential targets for retinal and retinal vascular disease types [35]. In addition, CD8^+^ T cells are implicated as a developmental factor for ROP progression [36]. We also report that AVR-123 significantly reduces dendritic cells. As previously mentioned, there is strong evidence that dendritic cell populations may be responsible for M2 (anti-inflammatory)-to-M1 (pro-inflammatory) macrophage transitions in certain retinopathies [69]. Increased dendritic cells, lymphocytes, and monocytes have also been shown to be produced systemically and directly progress diabetic retinopathy [70]. AVR-123 treatment also reduced neutrophils in contrast to the hyperoxia groups, which is an essential finding, as neutrophil-to-lymphocyte ratios are a risk factor for ROP treatment, as described in a 220-infant cohort study [38]. Our report on AVR-123’s ability to reduce specific immune cell types clearly shows that it does not simply inhibit a TLR cascade but modulates the immune response, showing a clear and pronounced anti-ROP physiological effect.

Our current study’s limitations include not assessing neovascularization, angiogenesis, or inflammatory markers immediately after hyperoxia exposure in P12 pups or optimizing the formulation, dosing routes, or frequency regimens. This study also lacks in the evaluation of TLR2, TLR4, and inflammatory cytokines in activated microglia and astrocytes in the hyperoxic retina and after AVR-123 treatment, which will be investigated later using Western blotting and ELISA.

## 5. Conclusions

In conclusion, we report that AVR-123 is an immunomodulating small molecule drug with the potential to prevent and/or treat ROP in both the hyperoxia and hypoxia stages of the pathology. Our study indicates that AVR-123 NP eyedrops have no visually observable side effects in mouse eyes and are productive in reducing both vaso-obliteration and angiogenesis, along with cytotoxic CD8^+^ T cells, neutrophils, macrophages, immune cell types, and cytokines that cause inflammation exacerbating the ROP pathology. AVR-123 is easy to administer via eyedrops. It can potentially mitigate possible side effects from intravitreal injection or surgery or further retinal damage that can occur simply from the procedure(s).

## Figures and Tables

**Table 1:** List of primers used.

Gene	Forward Sequence	Reverse Sequence
IL-1β	GCACTACAGGCTCCGAGATGAAC	TTGTCGTTGCTTGGTTCTCCTTGT
IL-6	CAACGATGATGCACTTGCAGA	TGTGACTCCAGCTTATCTCTTGG
IL-10	TGCTAACCGACTCCTTAATGCAGGAC	CCTTGATTTCTGGGCCATGCTTCTC
VEGF	ATCATGCGGATCAAACCTCACC	GGCTTTGTTCTGTCTTTCTTTGGTC
TGF-β2	GCCCCTGCTGTACCTTCGT	TGCCATCAATACCTGCAAATCTC
IGF-1	CAGTTCGTGTGTGGACCAAG	GAGTCTTGGGCATGTCAGTG
TNFα	5′-GCCTCTTCTCATTCCTGCTTG-3	5′-CTGATGAGAGGGAGGCCATT-3
iNOS	5′-GTTCTCAGCCCAACAATACAAGA-3′	5′-GTGGACGGGTCGATGTCAC
β-actin	GGACTCCTATGTGGGTGACG	CTTCTCCATGTCGTCCCAGT

**Table 2:** List of antibodies used.

Antibody	Dilution	Catalog No.	Vendor
CD80-FITC	1:200	Cat# 557226	BD Biosciences, San Jose, CA, USA
CD86-FITC	1:200	Cat# 555658	BD Biosciences, San Jose, CA, USA
MHC II-APC-Cy7	1:400	Cat# 56-5321-80	eBioscience, Waltham, MA, USA
CD4-PE-Cy7	1:200	Cat# 100526	Biolegend, San Diego, CA, USA
CD8-PE-Cy7	1:200	Cat# 100758	Biolegend, San Diego, CA, USA
B220-FITC	1:50	Cat# 553088	BD Biosciences, San Jose, CA, USA
CD3-PE	1:200	Cat# 555275	eBiosceince, Waltham, MA, USA
F4/80-APC	1:200	Cat# 50-112-9524	eBiosceince, Waltham, MA, USA
LY6G-PE-Texas Red	1:400	Cat# 50-112-4886	eBioscience, Waltham, MA, USA
CD11b-FITC	1:200	Cat# 562793	BD Biosciences, San Jose, CA, USA
CD11c-FITC	1:100	Cat# 561355	BD Biosciences, San Jose, CA, USA

## Data Availability

The data presented in this study are available on request from the corresponding author.

## Supplementary Materials

The following supporting information can be downloaded at: https://www.mdpi.com/article/10.3390/cells13161371/s1, Figure S1: Docking of AVR-121 with TLR4 (2z64, pdb) crystal structure; Table S1: Physicochemical properties of AVR-121 and AVR-123 compounds.
