# Supplementary material for: Novel Small Molecules with Anti-Inflammatory and Anti-Angiogenic Activity in a Mouse Model of Oxygen-Induced Retinopathy
Source: Cells. 2024 Aug 17;13(16):1371. doi: 10.3390/cells13161371 (PMC11353024; doi:10.3390/cells13161371)
Supplement: Supplementary file 1 [file cells-13-01371-s001.zip › cells-3135231-supplementary.pdf]

Supplemental Section

Novel small molecules with anti-inflammatory and anti-angiogenic activity in a mouse model of oxygen-induced retinopathy.

Adam S. Dayoub [1], Eesha Acharya,[1] Adnan Dibas[2]. Harlan Jones[2], and Suchismita Acharya [1,2*]

[1] AyuVis Research Inc, Fort Worth, TX, 76107, USA; adayoub@ayuvis.com; eacharya@ayuvis.com
[2] The North Texas Eye Research Institute, University of North Texas Health Science Center, Fort Worth, TX 76107, USA
* Correspondence: sacharya@ayuvis.com

Table S1: Physicochemical properties of AVR-121 and AVR-123 compounds

Compound ID	Chemical Structure	Molecular Weight (g/mol)	ClogP	tPSA	Aqueous Solubility	Aqueous Stability (25°C)
AVR-121	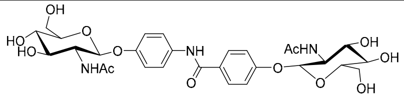	635.62	-0.43	245.6	3mg/mL	>1 months
AVR-123	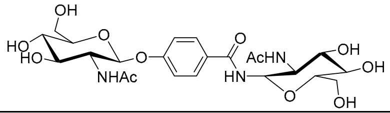	543.53	-0.93	236.37	3mg/mL	>1 months

ClogP: calculated log of partition coefficient; tPSA: total polar surface area

1. Preliminary Docking study of AVR-121 with TLR4 protein

Compounds AVR-121 and AVR-123 are chemical derivatives of chitohexaose and contain two N-acetyl glucosamine (NAG) moieties joined with hydrophobic linkers (**Table-1**). Previously, Panda et al. reported that chitohexaose (ing six NAG units via 1-4 beta linking) has a strong binding affinity to TLR4, and the docking experiment showed the ligand binds to an allosteric site (middle of the protein) and not the canonical LPS/MD2 binding pocket.[1] Based on this result, we screened our novel chitohexaose-based derivatives containing 1 to 5 NAG units attached in different ways.

Using AutoDock vina 1, the ligands were docked on three search boxes to ensure all of the areas of TLR4 protein were covered. Modifications on protein in the 2z64 crystal structure were retained, and 20 poses were generated in each experiment: LPS binding site, middle of the protein, and dimerization site of the

monomers, which makes 60 poses for each ligand. Ligands were set to be flexible, and the protein was fixed. Based on the three energy minimized poses summarized in Figure S1 (right bottom), we showed that compound AVR-121 prefers to spend more binding/resident time at the TLR4 protein dimerization site (red and green, minimum energy state) than the canonical LPS binding site (MD2 pocket) shown as the blue ligand. Size seems to be important for better affinity. In summary, this docking experiment with multiple ligands showed that adding bigger hydrophilic functional groups s to improve binding to TLR4, while adding hydrophobic parts does not have much effect. More detailed studies will be conducted in the future.

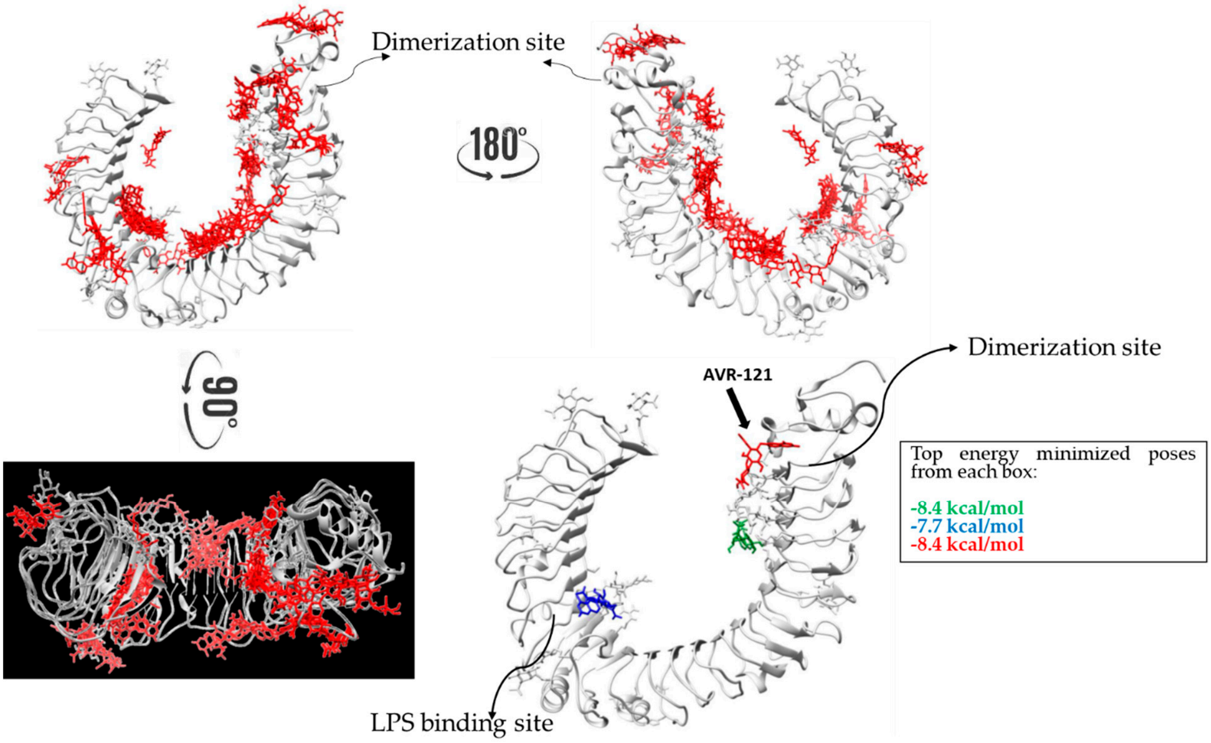

Supplemental figure S1: Docking of AVR-121 with TLR4 **(**2z64, pdb) crystal structure

1. Panda, S.K., et al., *Chitohexaose activates macrophages by alternate pathway through TLR4 and blocks endotoxemia.* PLoS Pathog, 2012. **8**(5): p. e1002717.
